# Comparison of different fibula procedures in tibiotalocalcaneal arthrodesis with a retrograde intramedullary nail: a mid-term retrospective study

**DOI:** 10.1186/s12891-023-07025-1

**Published:** 2023-11-13

**Authors:** Wenbao He, Haichao Zhou, Zhendong Li, Youguang Zhao, Jiang Xia, Yongqi Li, Cheng Chen, Hui Huang, Yi Zhang, Bing Li, Yunfeng Yang

**Affiliations:** 1 Department of Orthopedics, Shanghai Tongji Hospital, School of Medicine, Tongji University, Shanghai, 200065 China; 2 Department of Orthopaedics, Ruijin Hospital, Shanghai Jiao Tong University School of Medicine, Shanghai, China

**Keywords:** Orthopedic procedure, Osteoarthritis, Surgical technique, Talocalcaneal joint, Tibiotalar joint

## Abstract

**Background:**

Tibiotalocalcaneal (TTC) arthrodesis with a retrograde intramedullary nail for severe tibiotalar and talocalcaneal arthritis has a high fusion rate; however, no studies have focused on how to handle the fibula intraoperatively to achieve better results. This study aimed to compare the efficacies of various fibular procedures.

**Methods:**

We retrospectively reviewed the cases of severe tibiotalar and talocalcaneal arthritis in adults treated with TTC arthrodesis using a retrograde intramedullary nail between January 2012 and July 2017. The patients were divided into three groups according to different fibular procedures: Fibular osteotomy (FO), fibular strut (FS), and fibular preservation (FP). Functional outcomes and pain were assessed using the American Orthopedic Foot and Ankle Society (AOFAS) ankle and hindfoot score and visual analog scales (VAS), respectively. The operation time, fusion time, radiographic evaluation, and complications were also recorded.

**Results:**

Fifty-eight patients with an average age of 53.2 (range, 32–69) years were enrolled in the final analysis. The numbers of patients enrolled in the three groups were 21, 19, and 18 in the FO, FS, and FP groups, respectively. The mean postoperative follow-up time was 66.0 (range, 60–78) months. All groups showed a high fusion rate (90.5% for FO, 94.7% for FS, and 94.4% for FP) and significant improvement in AOFAS ankle and hindfoot scores and VAS scores at the latest follow-up. There were no significant differences in these parameters among the three groups. The mean operation time of FS (131.3 ± 17.1 min) was longer than that of FO (119.3 ± 11.7 min) and FS (112.2 ± 12.6 min), but the fusion time was shorter (15.1 ± 2.8 weeks for FS, 17.2 ± 1.9 weeks for FO, and 16.8 ± 1.9 weeks for FP). Statistically significant differences were observed in these parameters.

**Conclusions:**

TTC arthrodesis using a retrograde intramedullary nail is an effective procedure with a high rate of fusion to treat severe tibiotalar and talocalcaneal arthritis in adults; however, FSs can shorten fusion time when compared with FO and FP.

**Level of clinical evidence:**

Level 3.

## Introduction

Multiple etiologies have been proposed for severe tibiotalar and talocalcaneal arthritis in adult, including neurological, traumatic, hematological, and congenital causes [1–3], and is challenging for orthopedic surgeons. Various causes of ankle instability increase the contact stress on the ankle joint, leading to cartilage degeneration and ultimately severe tibiotalar and talocalcaneal arthritis, if not treated in a standardized manner [4]. Patients experience severe pain and have difficulty walking over time. Conservative methods are often futile, and once secondary degenerative changes and arthritis develop, arthrofusion is recommended [5].

Tibiotalocalcaneal (TTC) arthrodesis was first described in 1906 and some cases have been reported to be efficacious since then [6–10]. This is a joint sacrificial procedure for patients with severe tibiotalar and talocalcaneal arthritis that can create a stable, painless, and plantigrade foot. Various surgical techniques include blade plates, external fixators, and cancellous screws [5, 11–13]. There have been several studies on TTC arthrodesis [14–16]; however, to the best of our knowledge, none have focused on how to handle the fibula intraoperatively to achieve better results. Fibular osteotomy (FO) [15], fibular strut (FS) [17], and fibular preservation (FP) [13] are likely to be more feasible than other treatment options; however, the relative results remain unknown. This study aimed to evaluate the effects of fibular procedures during TTC arthrodesis using retrograde intramedullary nails in adults.

## Patients and methods

We retrospectively reviewed severe tibiotalar and talocalcaneal arthritis in adults treated with TTC arthrodesis using a retrograde intramedullary nail between January 2012 and July 2017 at our hospital. The inclusion criteria were as follows: ① Patient ages ranging from 18 to 70 years old, ② TTC arthrodesis by retrograde intramedullary nail, ③ various causes (including trauma, polio, charcot-Marie-Tooth, and unexplained clubfoot) resulting in severe tibiotalar and talocalcaneal arthritis that seriously affects the patient quality of life, and ④ follow-up time ranging from 60 to 80 months. The exclusion criteria were as follows: ① Patients with underlying diseases such as diabetes, immune system disorders, and blood disorders that affected bone healing; and ② patients who did not undergo follow-up regularly after surgery or whose follow-up visits were too infrequent, with fewer than three visits. The patients were divided into three groups according to different fibular procedures: Fibular osteotomy (FO), fibular strut (FS), and fibular preservation (FP). Anterior-posterior and lateral radiographs of the ankle and 3-dimensional reconstructions of computed tomography (CT) images were performed before the operation. All the surgeries were performed by the same group of surgeons. Preoperative and postoperative functions were evaluated using the visual analog scale (VAS) and the American Orthopedic Foot and Ankle Society (AOFAS) ankle and hindfoot scores [18, 19]. The operation time, fusion time, radiographic evaluation, and complications were recorded. This study was approved by the ethics committee of our hospital (K-W-2020-003).

### Operative procedure

Prophylactic intravenous antibiotics were administered before surgery. The patients were placed in the supine position. Surgery was performed under general anesthesia using a tourniquet on the thigh.

#### FO

**Fig. 1:**
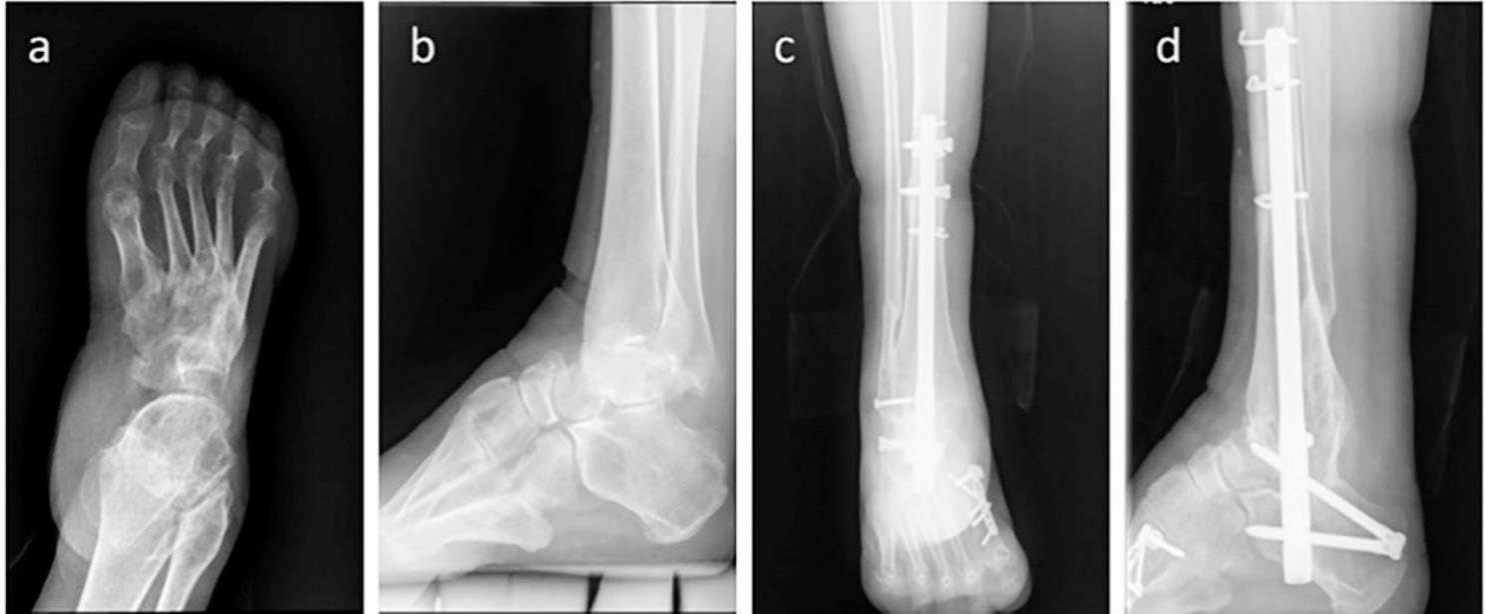
(a) Postoperative AP X-ray of the ankle of one patient in FO group. (b) Lateral X-ray of the patient. (c) Postoperative AP X-ray of the ankle of one patient in FP group. (d) Lateral X-ray of the patient

A 14-cm lateral approach centered on the fibula was used. The fibula was osteotomized approximately 5 cm above the prominence of the lateral malleolus. The distal tibiofibular syndesmosis was detached, and the cancellous bone of the distal fibula was used as a bone graft. The ankle joint was then exposed, adequate preparation of the tibial and talar articular surfaces was performed, and aggressive debridement was performed with the extraction of all residual cartilage and hyperplastic osteophytes. After thorough irrigation with saline solution, the subchondral bone of the tibiotalar joint was treated for microfractures. In addition, osteotomy of the medial malleolus was required to remove the residual cartilage thoroughly, correcting for talar rotation. Kirschner wires were inserted to temporarily maintain the position. X-ray images were obtained repeatedly to ensure satisfactory alignment. The tibia, talus, and calcaneus were reamed using a guide pin and an intramedullary nail (Smith & Nephew, Andover, MA, U.S.A.) was inserted under fluoroscopic guidance. The tip of the nail was impacted within the calcaneus and locked. Suction drains were placed, and the skin subcutaneous tissues were sutured in compliance with anatomy (Fig. 1a and b).

#### FP

An anterolateral approach was performed after tibial and talar articular surface procedures, aggressive debridement, and reduction of the talus, ankle, and subtalar joints were fixed using intramedullary nails (Fig. 1c and d).

#### FS

The same surgical approach and technique were used, and the fibula was osteotomized approximately 5 cm above the tip of the lateral malleolus. The distal portion was split in the sagittal plane, taking care to preserve its vascularity and soft tissues. The medial half was used as a bone graft, whereas the lateral half lagged behind the tibia and talus, serving as a lateral stabilizing strut. The medial half of the fibula was used for bone grafting, and the lateral fibular segment was then attached to the distal tibia and talus with two 4.0-mm cannulated screws directed laterally to medially. All other procedures were performed as previously described.

Additionally, the hindfoot alignment parameters were 5º of heel valgus, 5º to 10º foot external rotation, and 90º dorsiflexion. It was important to evaluate the first ray with a loading test after the above procedures. There was a need for dorsiflexion osteotomy of the first metatarsal if the first ray was still plantarflexed, which was aimed at an even pressure distribution.

### Postoperative management

Postoperatively, each patient was cast on a short-leg plaster slab. The affected limb was elevated to reduce swelling. Active motion of the toes was encouraged approximately 24 h after surgery. The suction drains were removed when the liquid inside was less than 10 ml per day. The dressing was changed every two days until the wound healed. We recommended outpatient follow-up with CT scans at 6, 12, and 24 weeks, and 1 year postoperatively. Plaster fixation was stopped after 6 weeks, and patients were allowed to wear walker boots to begin partial weight-bearing, which was beneficial for early functional exercise. The patients could perform full weight-bearing exercises 3 months postoperatively and resumed their preoperative activities 6 months postoperatively. Radiographic imaging, fusion time measurement, and follow-up functional recovery examinations were performed postoperatively. The results of the fusion were evaluated based on X-ray and CT images obtained at the patients’ follow-up visit, and interpretation of the imaging findings was performed by the same team of experienced radiologists and foot and ankle surgeons. The criteria for fusion according to CT or radiography are disappearance of the fracture line, formation of a bone scab at the surgical site, and formation of synostosis at the joint site. In addition, foot and ankle surgeons performed physical examinations to further corroborate the imaging findings. The AOFAS ankle and hindfoot scores and VAS scores were used to evaluate functional recovery at the final follow-up.

### Statistical analysis

Measurement data displayed normal distribution, and the homogeneity of variance was expressed as ${\bar x}$± s. The paired *t* test was used to compare pre- and post-operative data, and comparisons between groups were made using analysis of variance (ANOVA) or the chi-square test. An independent statistician performed the analyses, and all statistical analyses were performed using the Statistical Package for the Social Sciences (SPSS v19.0, IBM Corp., Armonk, NY, USA).

## Results

**Fig. 2:**
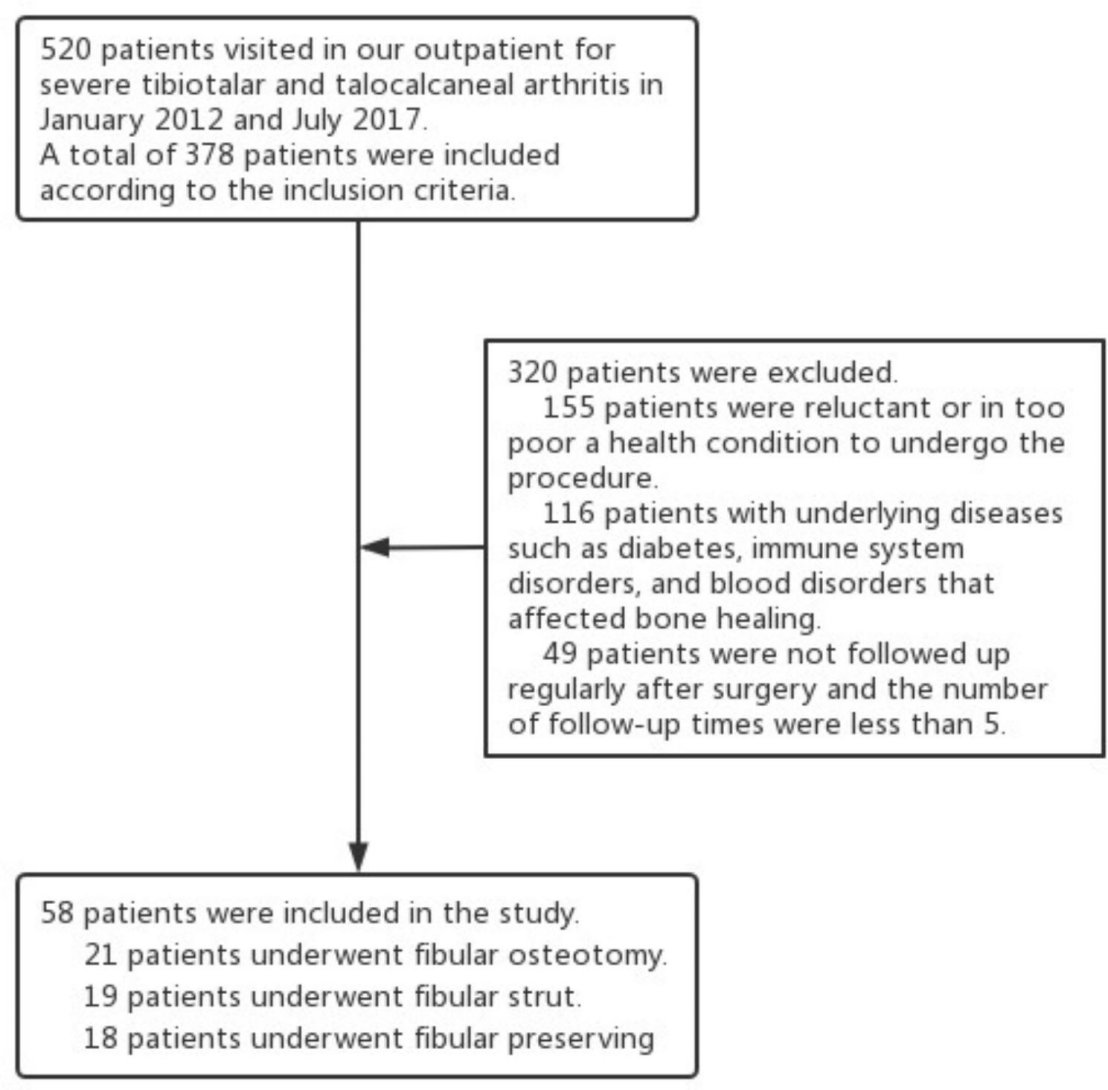
Study populartion

A total of 58 patients (including 29 males and 29 females) aged 53.2 ± 8.9 years (from 33 to 69 years) (Fig. 2) were included. All participants were divided into three groups by different fibular procedures, including FO (n = 21), FS (n = 19), and FP (n = 18) (Table 1). All 58 patients were followed up with for an average of 66.0 (range, 60–78) months, and there was no statistical difference in baseline information (Table 2). All groups required bone grafting at the fusion site and showed a high fusion rate, which was similar to the results of previous studies (FO: 90.5%, FS: 94.7%, FP: 94.4%), and after conservative treatment or secondary surgery, non-fused patients were able to live a normal life. AOFAS ankle and hindfoot scores at preoperative visits and final follow-up showed significant statistical difference in all three groups (FO: from 36.6 ± 9.1 to 77.0 ± 7.6, FS: from 34.4 ± 10.4 to 79.5 ± 5.7, and FP: from 37.7 ± 7.6 to 78.3 ± 5.5), but there were no statistical differences in the comparisons between groups. The change in the VAS score was similar to that in the AOFAS ankle and hindfoot scores. The mean operation time of FS (131.3 ± 17.1 min) was longer than that of FO (119.3 ± 11.7 min) and FS (112.2 ± 12.6 min) but the fusion time was shorter (FS: 15.1 ± 2.8 weeks, FO: 17.2 ± 1.9 weeks, and FP: 16.8 ± 1.9 weeks) (Table 2). They were able to walk normally, but had difficulty running.

**Table 1:** General conditions in three groups of patients

group	age	n	gender		side		etiology				follow-up (month)
			M	F	L	R	Neurogenic	Trauma	Poliomyelitis	Neglected idiopathic clubfoot	
FO	53.1 ± 9.3	21	10	11	11	10	4	4	7	2	64.9 ± 5.0
FS	52.6 ± 8.4	19	11	8	11	8	3	2	7	2	65.7 ± 5.8
FP	54.0 ± 9.1	18	8	10	9	9	4	5	5	2	67.6 ± 4.6
F/χ2	0.086		0.744		0.247		1.995				1.334
*p*	0.918		0.691		0.884		0.951				0.272

**Table 2:** Comparison of surgical parameters and functional evaluation in three groups

group	operation time (min)	fusion time (week)	fusion rate	AOFAS				VAS			
				pre-op	post-op	t	*p*	pre-op	post-op	t	*p*
FO	119.3 ± 11.7	17.2 ± 1.9	90.1%	36.6 ± 9.1	77.0 ± 7.6	16.334	<0.001	7.0 ± 1.4	1.7 ± 1.2	16.438	<0.001
FS	131.3 ± 17.1	15.1 ± 2.8	94.7%	34.4 ± 10.4	79.5 ± 5.7	19.739	<0.001	6.9 ± 1.3	1.6 ± 1.1	14.738	<0.001
FP	112.2 ± 12.6	16.8 ± 1.9	94.4%	37.7 ± 7.6	78.3 ± 5.5	21.567	<0.001	7.0 ± 1.3	1.9 ± 1.0	14.541	<0.001
F/χ^2^	8.912	5.256	0.549	0.851	0.453			0.028	0.182		
*p*	p<0.001	0.008	1.000	0.432	0.638			0.972	0.834		

**Fig. 3:**
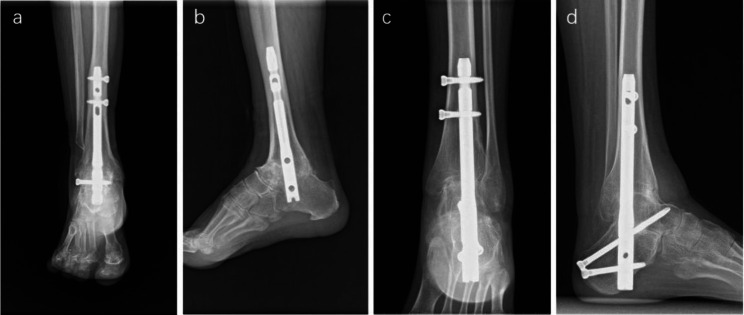
(a) Preoperative AP X-ray of the foot of the patient who experienced the tibial split in FS group. (b) Lateral X-ray of the patient. (c) AP X-ray of the patient at the latest follow-up. (d) Lateral X-ray of the patient. The patient was satisfied with the procedure

One patient in the FS group experienced a tibial split during the operation; therefore, we used a longer intramedullary nail and fixed the fracture with three steel wires. The patient was informed that early weight-bearing should be avoided. Fortunately, the fracture healed and joint fusion was achieved 22 weeks after surgery. At the final follow-up, the patient did not complain of any discomfort (Fig. 3a, b, c and d). No severe postoperative complications, such as periprosthetic fracture, hardware failure, or full-thickness wound infection, were observed in the present study. None of the patients showed signs of deformity recurrence at the most recent follow-up.

## Discussion

In this study, we compared the therapeutic effect of three different fibula management groups (FO, FS and FP) in a sample of 58 patients treated with TTC fusion. The results indicated that TTC arthrodesis using a retrograde intramedullary nail was an effective procedure with a high rate of fusion to treat severe tibiotalar and talocalcaneal arthritis in adults. And notably, FS, which could shorten fusion time when compared with FO and FP, seemed like a more feasible choice in the TTC arthrodesis surgery.

First of all, how effective is the TTC arthrodesis procedure? Indications for TTC arthrodesis include ankle and subtalar joint arthritis, severe acute trauma such as pilon fracture, osteonecrosis of the talus, severe hindfoot deformity, and failed total ankle replacement [20–22]. Persistent pain seriously affects patients’ daily lives and often requires surgery as the disease progresses [23]. It has been reported to effectively improve pain, correct deformities, and restore ankle and hindfoot stability, even in patients with Charcot neuroarthropathy of the hindfoot [24–26]. In this study, the average AOFAS ankle and hindfoot scores and the VAS score at the last follow-up were 78.0 and 1.8 respectively, which are similar to the results of previous studies and owing to the disappearance of sagittal and hindfoot motion, the AOFAS ankle and hindfoot scores rarely exceed 85 points [5]. Theoretically, when compared to ankle arthrodesis, the function and satisfaction of the ankle and hindfoot should be worse because TTC leads to complete stiffness of the ankle and hindfoot [27]. However, Ajis et al. [7] compared patients who used the two fusion methods, and the results showed that there was no significant difference in postoperative pain, patient satisfaction, and daily function recovery. Chopra et al. [28] demonstrated that TTC arthrodesis was not more detrimental to gait mechanics than ankle arthrodesis. Consistent with the results of previous studies, all patients in the present study were satisfied with their postoperative outcomes. Such patients adapt to poor joint function for a long time prior to surgery, and retaining or not retaining the subtalar joint, which is a series of micromotor joints, has little impact on their daily lives. The elimination of pain and stabilization are the primary patient requirements. Collectively, TTC arthrodesis was confirmed to have a satisfactory clinical efficacy.

In addition, the choice of internal fixation devices is also an important factor affecting the outcome of TTC arthrodesis. Improving joint fusion rates, stabilizing features, and reducing complications are essential [29].Three main types of internal fixation are used for TTC: screws, plates, and retrograde intramedullary nails. Headless compression screws can also be used for TTC arthrodesis with minimal incision, short operation times, low incidence of complications, and good postoperative outcomes [30]. However, Hamid et al. indicated that plates and intramedullary nails could provide equal compressive forces at the ankle and subtalar joints, both of which were better than screw-only constructs [31]. Biomechanical studies have also shown that blade plate fixation is a static device that cannot provide stress loading as in intramedullary nail fixation [32, 33]. In this study, patients were allowed to start weight-bearing training early after the operation, which could significantly shorten the bed rest time and improve satisfaction in patients with first-stage bilateral fusion. None of the patients had loose or broken internal fixations, which may occur in the case of plate fixation. Therefore, compared to the eccentric fixation of the plate, TTC arthrodesis with a retrograde intramedullary nail is an axial fixation, and the alignment is extremely stable. However, because the procedure is more invasive, TTC with retrograde intramedullary nail fixation has a higher rate (> 50%) of surgical complications [34–38]. Therefore, it needs to be emphasized that intramedullary nail fixation is more technically demanding for the operator, and attention should be paid to avoid intraoperative complications, such as lateral peroneal nerve and vascular injury or fractures. Fortunately, this study indicated a complication rate of 12.1%, including delayed healing of the incision (three patients), mild wound infection (three patients), and intraoperative medically induced fractures (one patient), with effective recovery. Generally, TTC arthrodesis with a retrograde intramedullary nail is a reliable treatment.

Although TTC arthrodesis using a retrograde intramedullary nail is a well-established procedure, the best option as a fibular procedure remains uncertain. All of FO, FS and FP have been reported in the existing literature, but no one has focused whether the choice of different fibula management would make a difference in TTC arthrodesis surgical outcomes [13, 15, 17]. Shah et al. introduced a surgical procedure that involves inserting a FS graft intramedullary using adjuvant hardware fixation, reporting that the FS was beneficial to bone fusion and the intraoperative use of autologous bone grafts helped reduce infection rates [29]. In this study, patients in the FS group had a significantly shorter fusion time than those in the other two groups, and the lateral support of the fibula provided good anti-rotation ability for the tibiotalar and subtalar joints and enhanced their stability. The preserved lateral half of the fibula has a good blood supply, which is often used for free vascularized fibular grafting to treat osteonecrosis of the femoral head because the blood supply is particularly abundant in the middle and lower thirds of the fibula [39–41]. The medial side is a cancellous bone surface, which can be used as a good bone graft material. Osteotomy can create a situation similar to that occurring during fracture healing, which leads to an increase in bone metabolism that creates an increase in blood flow to the site. This may explain the shorter fusion time observed in the FS group. Furthermore, the FS avoids the use of autogenous or allogeneic iliac bone grafts. Using this method, Akra et al. [42] believed that the fusion rate could reach 100%, which is consistent with the results of this study. Notably, although patients with severe tibiotalar and talocalcaneal arthritis showed great satisfaction using FSs, we recommend that this procedure should not be performed too early in general because an abnormal stress condition for a long time may lead to degenerative changes in the adjacent joints. Therefore, if TTC arthrodesis using a retrograde intramedullary nail is indicated, a FS is recommended to shorten the fusion time.

This study has some limitations. First, the sample size was small, and a large-scale, long-term, prospective, randomized study is needed to verify the results. Second, the efficacy of TTC arthrodesis using retrograde intramedullary nails in patients with underlying diseases that affect bone healing is unknown. Third, although it has been reported that using weightbearing CT (WBCT) in deformity and osteoarthrosis assessment pre- and post-operatively has potential benefits [43, 44], no WBCT scans were performed in this study because of the lack of equipment. WBCT should be used in more studies if possible. Finally, some social factors, such as smoking, alcohol consumption, and living environment, were not excluded from influencing treatment outcomes. Recently, some new treatment methods, such as minimally invasive surgery for TTC arthrodesis using a retrograde intramedullary nail [45] and TTC arthrodesis with the Paragon28 Silverback™ plating system [21], have been reported to achieve good therapeutic results in patients with severe tibiotalar and talocalcaneal arthritis and deformities of the ankle and hindfoot. Owing to the lack of relevant controlled clinical studies, future large-sample randomized controlled studies with more stringent control variables are needed to confirm the best surgical procedure for TTC arthrodesis.

## Conclusion

According to the results of this study, TTC arthrodesis by retrograde intramedullary nailing is an effective procedure to treat severe tibiotalar and talocalcaneal arthritis in adults, as FSs can shorten the fusion time compared with FO and FP. Future research will need to expand the number of cases to further confirm the effectiveness of TTC arthrodesis using retrograde intramedullary nails with FSs, and then compare this procedure with other non-intramedullary nailing TTC procedures to explore the best options.

## Data Availability

The datasets supporting the conclusions of this article are all included within the article.
